# Gallbladder large cell neuroendocrine carcinoma in a patient with multiple myeloma: a case report and literature review

**DOI:** 10.3389/fonc.2025.1664914

**Published:** 2025-11-21

**Authors:** Yuhan Yin, Xinjie Dai, Dan Li, Xinyu Yang, Nan Ming, Kang Xiang, Dengding Wu, Kong Ai, Rucheng Yao, Jun Hu

**Affiliations:** 1 The First College of Clinical Medical Science, China Three Gorges University, Yichang, Hubei, China; 2 Department of Hepatobiliary Surgery, Yichang Central People’s Hospital, Yichang, Hubei, China; 3 Department of Hepatobiliary Surgery, The Second People’s Hospital of China Three Gorges University, The Second People’s Hospital of Yichang, Yichang, Hubei, China

**Keywords:** gallbladder large cell neuroendocrine carcinoma, GB-LCNEC, multiple myeloma (MM), neuroendocrine tumors (NENs), well-differentiated neuroendocrine neoplasms (NET), poorly differentiated neuroendocrine carcinomas (NEC), mixed neuroendocrine-non-neuroendocrine tumors (MiNEN)

## Abstract

Gallbladder Large Cell Neuroendocrine Carcinoma (GB-LCNEC), an extremely rare pathological subtype of gallbladder malignancies, is characterized by high aggressiveness and insidious onset, often leading to poor prognosis and presenting significant clinical management challenges. This report presents the complete treatment course of a patient diagnosed with GB-LCNEC complicated by active Multiple Myeloma (MM), accompanied by a systematic review of relevant literature published over the past decade. Preoperative imaging revealed a gallbladder mass, which was pathologically confirmed postoperatively as GB-LCNEC through histological and immunohistochemical analysis. The tumor demonstrated large cell morphology, high mitotic activity (Ki-67 index of 50%), and strong expression of neuroendocrine markers, including Insulinoma-Associated Protein 1 (INSM1). Despite the concurrent diagnosis of active MM, a Multidisciplinary Team (MDT) successfully performed radical resection for gallbladder carcinoma, which included cholecystectomy, wedge resection of adjacent hepatic tissue, and regional lymphadenectomy at the hepatic hilum. An R0 resection was achieved, laying a solid foundation for postoperative management. Given the patient’s limited tolerance to the standard Etoposide–Cisplatin (EP) chemotherapy regimen, a milder, individualized approach using the Capecitabine–Temozolomide (CAPTEM) regimen was selected. Simultaneously, the patient’s MM was treated with the Daratumumab–Lenalidomide–Dexamethasone (DRD) regimen. At six-month follow-up, there was no evidence of recurrence or metastasis, and the patient maintained a favorable general condition. This case underscores that in highly aggressive malignancies such as GB-LCNEC, surgical resection remains a cornerstone of effective disease control and survival extension, even in the presence of severe comorbidities. MDT-based decision-making and personalized treatment strategies are essential for optimizing therapeutic outcomes and minimizing treatment-related risks. Future research should prioritize the development of multicenter clinical registries and large-scale molecular profiling, while also evaluating emerging modalities such as targeted therapies and immunotherapy to ultimately improve the prognosis of this rare tumor entity.

## Introduction

Gallbladder cancer (GBC) is a rare malignant tumor of the biliary tract (1). Due to its atypical early symptoms, aggressive nature, and frequent metastasis upon detection, it carries an extremely poor prognosis with a 5-year survival rate below 5% (2). Among all gallbladder malignancies, neuroendocrine tumors (NENs) represent an exceptionally rare category, accounting for approximately 2.1%. According to the 2022 World Health Organization (WHO) classification of digestive system tumors, gallbladder neuroendocrine tumors are categorized into well-differentiated neuroendocrine neoplasms (NET), poorly differentiated neuroendocrine carcinomas (NEC), and mixed neuroendocrine-non-neuroendocrine tumors (MiNEN) (3). NEC is further divided into small cell neuroendocrine carcinoma (SCNEC) and large cell neuroendocrine carcinoma (LCNEC).

Gallbladder Large Cell Neuroendocrine Carcinoma (GB-LCNEC) is a rare subtype of neuroendocrine carcinoma with extremely high malignancy (4). Despite existing retrospective analyses, the global reporting of pure-type GB-LCNEC cases remains remarkably limited (5). Current data indicate that this disease predominantly affects middle-aged and elderly women aged 60–70 (6). Its clinical manifestations lack specificity, typically presenting with right upper abdominal dull pain, appetite loss, or weight reduction, making it difficult to distinguish from gallbladder adenocarcinoma or other biliary tract diseases (7). Imaging studies also lack distinctive diagnostic features, leading to challenges in preoperative diagnosis. Current diagnosis primarily relies on postoperative pathological results and immunohistochemical markers (8). The disease often shows rapid proliferation, local invasion, and even distant metastasis at early stages, severely limiting intervention opportunities (9). Multiple studies demonstrate that median overall survival (MOS) generally ranges from 6 to 12 months—significantly shorter than common types like gallbladder adenocarcinoma—indicating an extremely poor prognosis and highlighting the urgent need for heightened clinical awareness (10).

When primary malignant tumors of two distinct histological origins—collectively termed multiple primary malignancies (MPMN)—occur in the same patient, clinical decision-making becomes significantly more complex (11). For a hematologic malignancy patient diagnosed with multiple myeloma (MM) who concurrently develops GB-LCNEC—a highly rare solid tumor—clinicians face substantial challenges in determining surgical approaches, selecting chemotherapeutic agents, and managing drug toxicity. This unique clinical scenario has not been documented in prior literature, and the potential underlying pathogenic mechanisms warrant further investigation (12).

In this report, we present the complete diagnostic and therapeutic course of a newly identified case of gallbladder large cell neuroendocrine carcinoma (GB-LCNEC) in the context of ongoing diagnosis and treatment of multiple myeloma (MM). A comprehensive literature review is also included. This study aims to explore the clinicopathological characteristics and diagnostic challenges of this rare malignancy, with particular emphasis on how multidisciplinary collaboration can facilitate the development of individualized, integrated treatment strategies for patients with complex hematologic comorbidities. The goal is to provide valuable clinical insights for managing similarly challenging cases in future practice.

## Case report

A 56-year-old female patient was admitted to the hematology department of our hospital for treatment of multiple myeloma diagnosed more than two months earlier. During a routine abdominal ultrasound evaluation, an unexpected gallbladder space-occupying lesion was detected. Although the patient had a history of multiple gallstones, she reported no significant symptoms such as abdominal pain, bloating, or jaundice over the past six months, with only a non-specific weight loss of approximately 3 kg. Physical examination revealed no positive findings: the abdomen was flat and soft without tenderness or rebound tenderness, and no abnormal masses were palpable.

Upon admission, comprehensive diagnostic tests were conducted. Laboratory results showed: hemoglobin 84 g/L; albumin 36.3 g/L; immunoglobulin G (IgG) 41.40 g/L (reference range: 8.6–17.4 g/L); serum κ-light chain 0.55 g/L (reference range: 1.7–3.7 g/L); β2-microglobulin 2.59 mg/L (reference range: 0–2.1 mg/L). These parameters supported the diagnosis of multiple myeloma. Tumor marker levels, including carcinoembryonic antigen (CEA) and alpha-fetoprotein (AFP), were within normal ranges.

**Figure 1:**
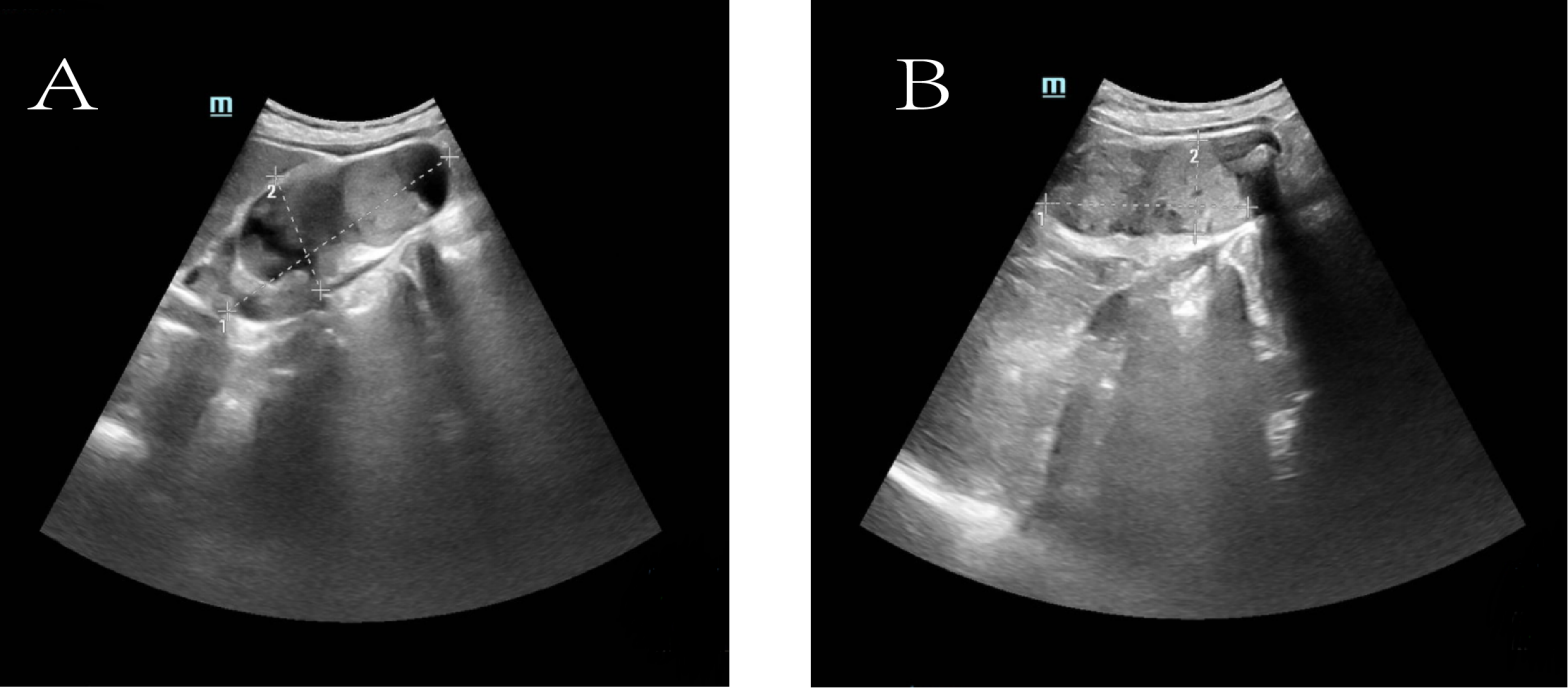
Ultrasonographic images of the gallbladder. **(A)** The gallbladder is enlarged (measuring approximately 9.7 × 4.2 cm) with a non-thickened wall (delineated by dashed line). Multiple hyperechoic foci are observed within the lumen, accompanied by dense posterior acoustic shadowing. **(B)** A medium-echoic mass, measuring approximately 7.0 × 3.1 cm, is visible within the gallbladder lumen (outlined by dashed line).

**Figure 2:**
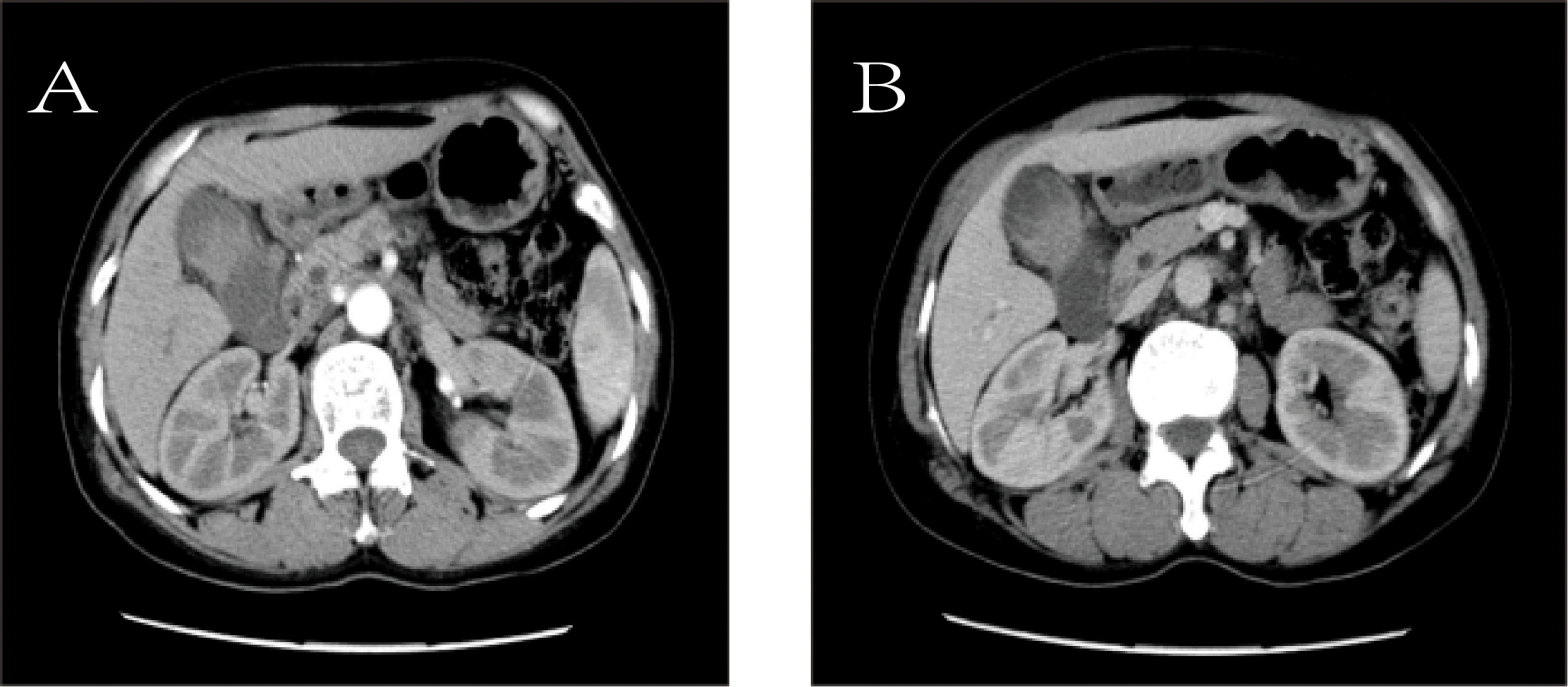
Contrast-enhanced computed tomography (CT). **(A)** Arterial phase image shows the mass (white arrow). **(B)** Venous phase image of the same mass (white arrow).

**Figure 3:**
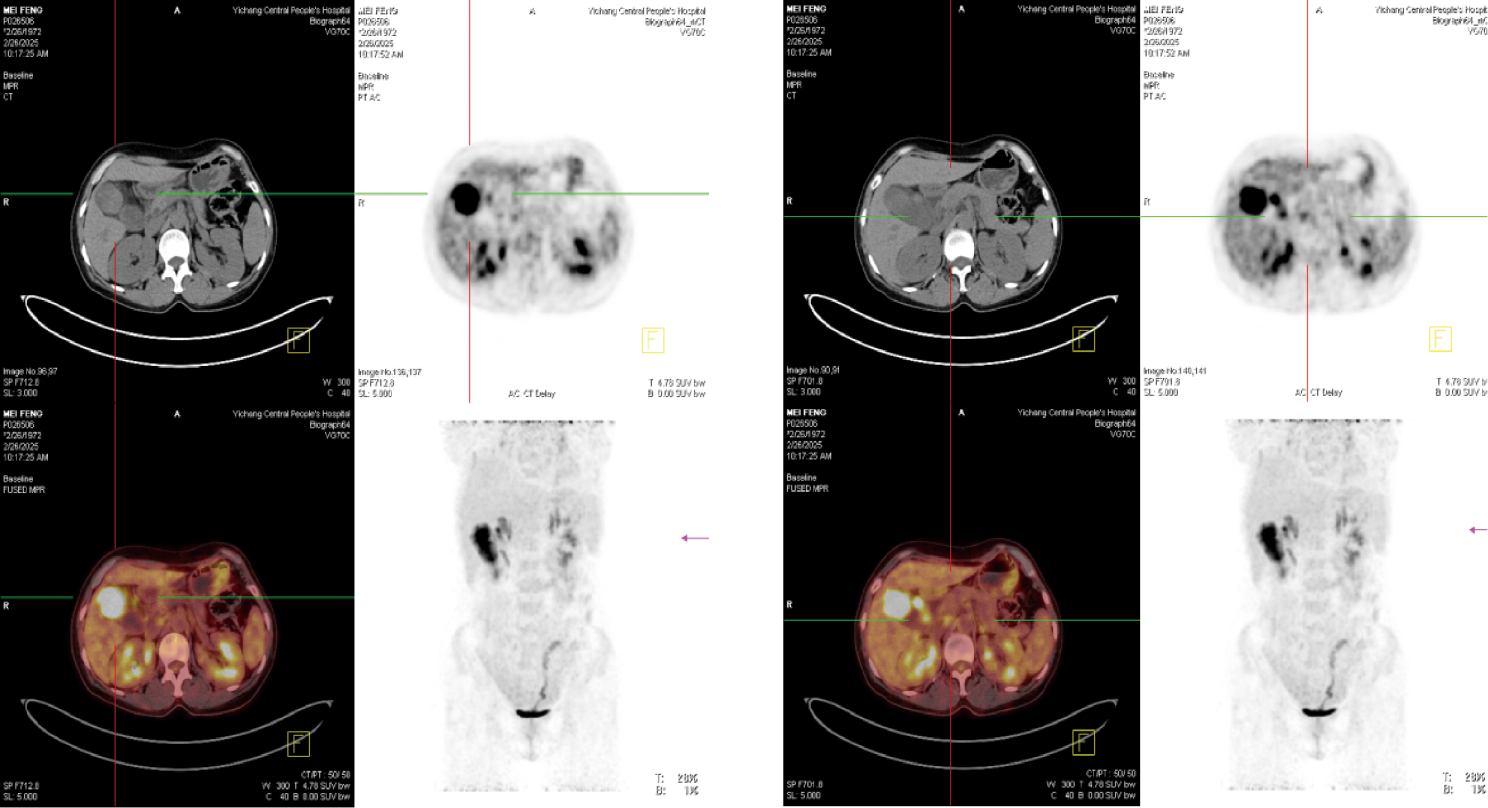
PET-CT.

To further clarify the nature of the gallbladder mass, abdominal color Doppler ultrasound, upper abdominal contrast-enhanced CT, and PET-CT examinations were performed. Ultrasound revealed an enlarged gallbladder measuring approximately 9.7 × 4.2 cm with a moderately echogenic nodule filling its lumen, of indeterminate nature (See **Figure 1**). Contrast-enhanced CT showed increased gallbladder volume with localized uneven wall thickening and soft tissue density nodules protruding into the lumen, strongly suggesting neoplastic growth (**Figure 2**). Multiple nodular shadows were observed in the hepatic portal region, likely representing enlarged lymph nodes, with the largest measuring 1.9 × 1.7 cm. PET-CT confirmed increased retroperitoneal lymph nodes with elevated SUVmax values of approximately 18.0 and 25.9 on early and delayed imaging, respectively (**Figure 3**). These findings, showing both gallbladder soft tissue nodules and enlarged lymph nodes with abnormal metabolic activity, led to a preoperative diagnosis of gallbladder carcinoma with hepatic portal lymph node metastasis.

Following preoperative evaluation and multidisciplinary team (MDT) discussion, the patient was scheduled for surgery. On March 6, 2025, under general anesthesia, laparoscopic exploration was initiated but converted to open surgery, and a radical cholecystectomy was performed, including gallbladder removal, hepatic wedge resection, and hilar lymph node dissection.

**Figure 4:**
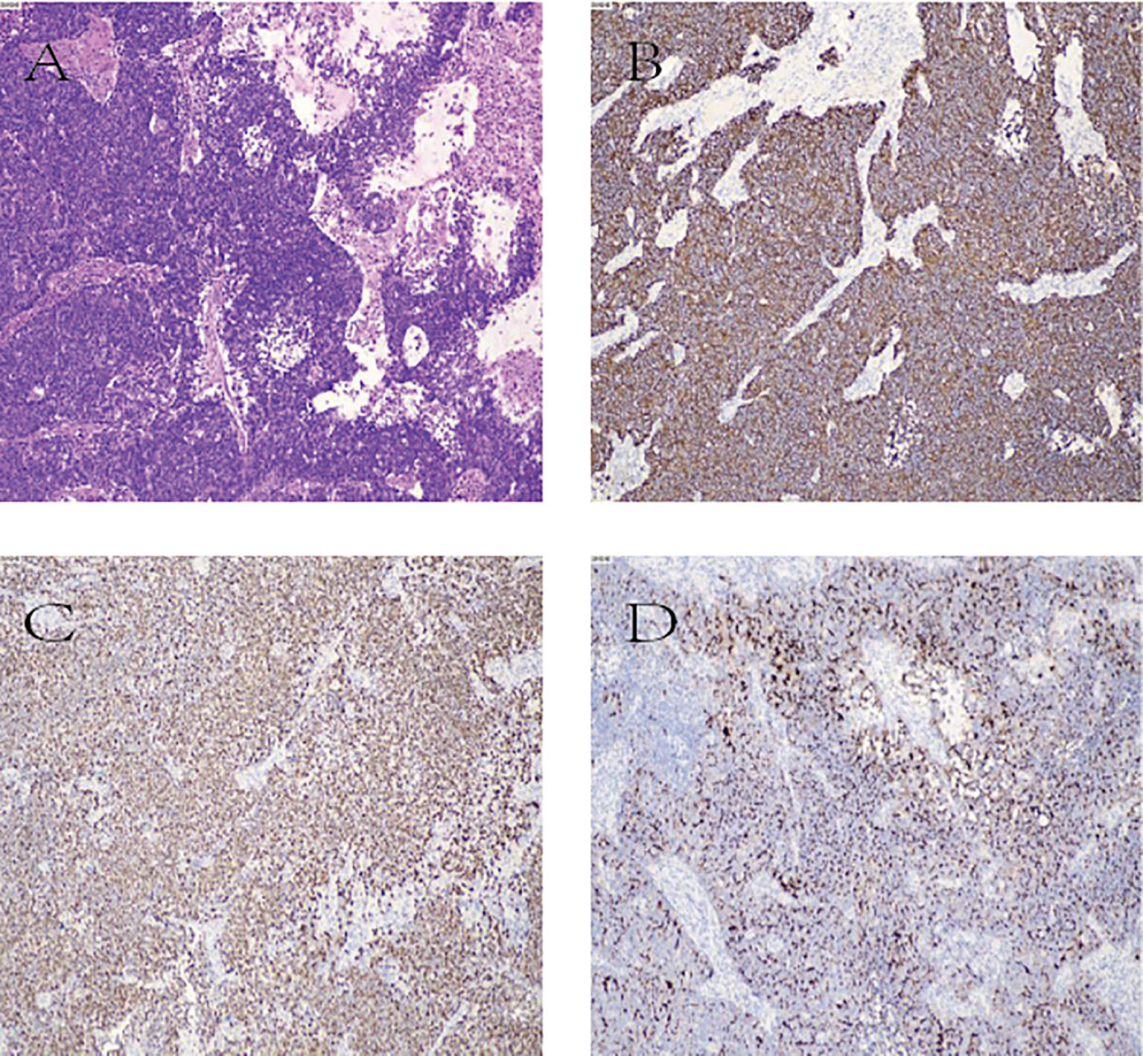
**(A)** Hematoxylin and eosin (H&E) staining image; **(B–D)** Immunohistochemical staining images. **(B)**: CK8 positive (CK8+); **(C)** INSM1 positive (INSM1+); **(D)** MUC1 positive (MUC1+).

Postoperative pathology revealed large cell neuroendocrine carcinoma (GB-NEC) of the gallbladder, maximum diameter 5.5 cm, invading surrounding connective tissue but without penetration of the serosa or invasion of the liver. Mitotic count exceeded 20 per 2 mm². Cancer invasion was seen in nerves and intravascular tumor thrombi; no invasion was found at the gallbladder neck margins. Six lymph nodes on the gallbladder serosal surface showed complete metastasis (6/6). Immunohistochemistry results were: CK7 (+), CK20 (focal +), CK8/18 (+), CK19 (+), MUC1 (paracellular punctate +), CDX-2 (+), SATB-2 (focal +), Ki-67 (LI ~50%), INSM1 (+), CD56 (focal +), p40 (–). Microscopic examination of partial liver tissue showed scattered infiltrating lymphocytes in interstitial and portal areas without cancer invasion. Hilar lymph nodes showed metastatic carcinoma (1/6). TNM pathological stage: pT2bN2Mx (**Figure 4**).

These findings ultimately confirmed the diagnosis of gallbladder large cell neuroendocrine carcinoma (GB-LCNEC), stage pT2bN2Mx. The presence of 6/6 metastatic serosal lymph nodes indicated tumor penetration through the gallbladder wall. However, preoperative and intraoperative assessments based on imaging and surgical exploration could not precisely determine the depth of invasion. Given the patient’s concurrent active multiple myeloma and poor bone marrow reserve, the MDT opted for a relatively conservative wedge resection strategy to minimize perioperative risks while still achieving R0 resection.

**Figure 5:**
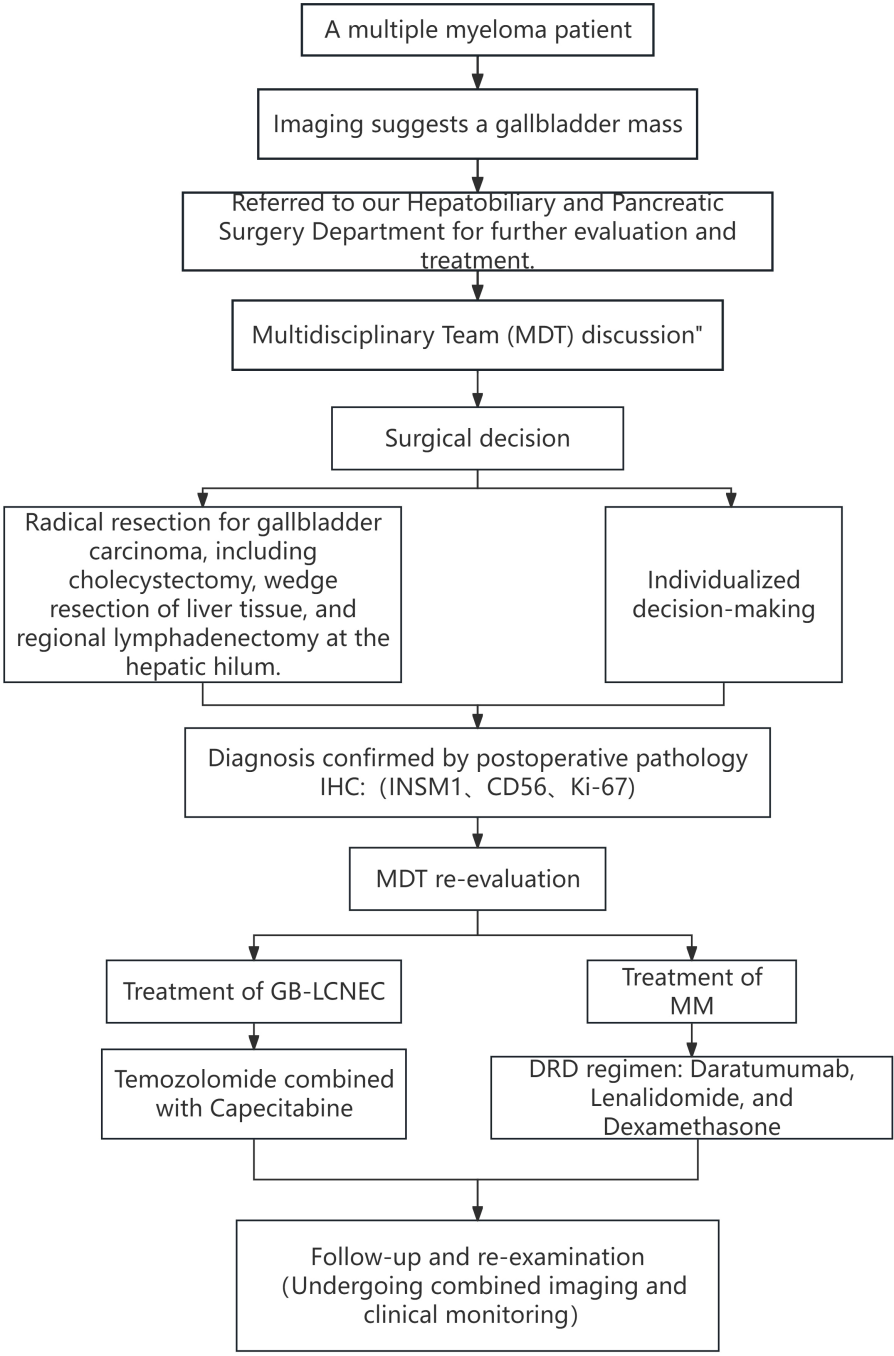
Diagnostic and therapeutic pathway for a patient with GB-LCNEC and multiple myeloma.

The patient recovered well postoperatively. Considering the dual malignancies, the MDT reconvened to formulate a comprehensive follow-up treatment plan. For GB-LCNEC, the standard EP regimen (cisplatin + etoposide) was considered inappropriate due to the high likelihood of intolerable bone marrow suppression and infection risks. After extensive MDT discussions, the patient was started on an oral chemotherapy regimen combining temozolomide with capecitabine (CAPTEM), which has relatively mild toxicity and is more suitable for patients with hematologic comorbidities. For multiple myeloma management, the patient had initially received the DVD regimen (daratumumab, bortezomib, dexamethasone) preoperatively and achieved partial response (PR). Subsequently, treatment was switched to DRD (daratumumab, lenalidomide, dexamethasone) to balance efficacy with tolerability. The detailed diagnostic and therapeutic workflow is shown in **Figure 5**.

At the time of this report, the patient had completed 6 months of follow-up with good general condition. A PET-CT scan on September 8, 2025 showed no recurrence or metastasis. Initially, the patient experienced significant psychological distress due to the coexistence of two malignancies, but both physical and mental status improved markedly following successful surgery and the “tailor-made” comprehensive treatment plan, with satisfactory outcomes reported.

## Discussion

Gallbladder high-grade neuroendocrine carcinoma, particularly the large cell subtype (GB-LCNEC), represents an extremely rare pathological entity among digestive system tumors, with its exact pathogenesis not yet fully elucidated (9). By combining a review of the literature with our clinical case, this study aims to explore the clinicopathological characteristics (8), diagnostic standards, therapeutic strategies, and management challenges of GB-LCNEC in patients with severe comorbidities (13).

There are currently three main hypotheses regarding the tissue origin of GB-LCNEC. The first suggests that GB-LCNEC may arise from argyrophilic cells or multipotent stem cells located in areas of intestinal metaplasia within the gallbladder mucosa, often associated with gallstones and chronic inflammation (14). Our patient’s history of gallstone disease supports the classic pathway of “chronic inflammation → metaplasia → dysplasia → carcinogenesis (15).” The second hypothesis posits that undifferentiated multipotent stem cells in gallbladder tissue undergo divergent differentiation (16). The third hypothesis proposes that GB-LCNEC may originate from transdifferentiation of pre-existing adenocarcinoma cells (3). This is supported by reports of cases where GB-LCNEC coexists with adenocarcinoma, forming mixed tumors (MiNEN). However, in the present case, no adenocarcinoma component was observed in postoperative pathological sections, suggesting a primary neuroendocrine carcinoma originating from neuroendocrine precursor cells.

To systematically evaluate the clinical features of GB-LCNEC, we reviewed and analyzed cases published in PubMed over the past decade that included relatively complete diagnostic, pathological, and therapeutic data (see **Table 1**) (1, 17–30). The findings highlight the complex clinical profile of this rare and aggressive tumor: it predominantly affects middle-aged and elderly women, and most patients present with lymph node or liver metastases at diagnosis, reflecting its stealthy and rapid progression. Some cases exhibit mixed histopathological patterns, often coexisting with adenocarcinoma but rarely with other extracholecystic malignancies, underscoring the rarity of this condition (17). Radical surgery remains the primary treatment, with some patients benefiting from adjuvant chemotherapy (e.g., EP or EC regimens), achieving survival up to three years. Nevertheless, many cases demonstrate rapid progression and death, reflecting the highly variable prognosis. Clinically, GB-LCNEC lacks specific symptoms and imaging features, making preoperative diagnosis nearly impossible. Thus, postoperative pathological examination remains the only reliable diagnostic approach, particularly immunohistochemical analysis. In addition to traditional neuroendocrine markers such as Synaptophysin (Syn) and Chromogranin A (CgA), this case also showed positivity for INSM1, a novel biomarker with high specificity that enhances diagnostic accuracy (30). The high Ki-67 index (50%) indicated extremely high proliferative activity at the molecular level, consistent with the tumor’s aggressive biological behavior (17). Moreover, inactivation of TP53 and RB1 genes has been frequently reported in high-grade NECs and is considered a critical molecular event driving loss of cell cycle control and hyperproliferation (1).

**Table 1:** A review of global case reports of gallbladder large cell neuroendocrine carcinoma over the past decade.

First author (year)	Country/region	Age/sex	Diagnosis	Tumor size (cm)	Lymph Node/Liver metastasis	Treatment	Survival	Article title	Combined with other tumors
Tingting Yu (2022) (17)	China	56/F	GB-LCNEC with adenocarcinoma of the gallbladder	3.2× 1.6	Yes/Yes	Radical resection of gallbladder carcinoma with regional lymphadenectomy	9 months (Alive)	Primary mixed large cell neuroendocrine carcinoma and adenocarcinoma of the gallbladder: A case report and literature review	Yes(Gallbladder adenocarcinoma
Dhruba Narayan Sah (2024) (18)	Nepal	65 /F	GB-LCNEC	7 x 6	Yes/Yes	Open extended cholecystectomy with adjacent liver resection	6 months (Alive)	Large Cell Neuroendocrine Carcinoma of Gallbladder: A Case Report	No
Shapera 2019 (19)	USA (Las Vegas)	65 /F	GB-LCNEC	2.5	No/No	Laparoscopic cholecystectomy+EP regimen (cisplatin + etoposide)	19 months (Alive)	Survival : a rare outcome in large cell neuroendocrine carcinoma of the gallbladder	No
Salvatore Buscemi(2015) (20)	Italy	76/F	GB-LCNEC	1.8	Yes/Yes	Laparoscopic cholecystectomy+EC regimen (cisplatin + epirubicin)	5 months (Deceased)	“Pure” large cell neuroendocrine carcinoma of the gallbladder. Report of a case and review of the literature	No
Samyak Dhruv (2023) (21)	America	72/M	GB-LCNEC	7.2 × 4.9	Yes/Yes	Palliative chemotherapy	Hospice care	Large Cell Neuroendocrine Carcinoma of the Gallbladder: Where Survival Is a Rare Entity – Case Report and Review of the Literature	No
Rodney E Shackelford (2022) (22)	America	62/M	GB-LCNEC	3	Yes/No	Laparoscopic cholecystectomy	Lost to follow-up	A Rare Case of Pure Primary Large Cell Neuroendocrine Carcinoma of the Gallbladder	Yes(Breast cancer)
Saamia Shaikh (2023) (23)	America	73/F	GB-LCNEC	2.0×1.7×1.0	Yes/No	Laparoscopic cholecystectomy+EP regimen (cisplatin + etoposide)	3 years (Alive)	A rare occurrence of a poorly differentiated large cell neuroendocrine carcinoma of the gallbladder: A case report and review of the literature	No
Anton Fick (2023) (24)	South America	65/F	GB-LCNEC with adenocarcinoma of the gallbladder	3.2	No/No	Laparoscopic cholecystectomy with hepatic wedge resection of segments 4B/5 and portal lymph node dissection, followed by adjuvant chemotherapy with Capecitabine.	3 years (Alive)	An Unusual Case of Large Cell Neuroendocrine Cancer of the Gallbladder With Mixed Adenocarcinoma Component in a Patient With Pancreatobiliary Maljunction	Yes(Gallbladder adenocarcinoma)
Sarthak Soin (2018) (25)	America	62/F	GB-LCNEC with adenocarcinoma of the gallbladder	5×4×3	No/No	Radical cholecystectomy with partial hepatectomy and regional lymph node dissection+EP regimen (cisplatin + etoposide)	2 months (Deceased)	Large cell neuroendocrine carcinoma and adenocarcinoma of gallbladder with concomitant hepatitis C infection	Yes(Gallbladder adenocarcinoma)
Ahmad Abutaka (2019) (26)	Qatar	67/F	GB-LCNEC		Yes/Yes	Laparoscopic cholecystectomy+Central hepatectomy with wedge resection of an intraoperatively discovered lesion in segment III, and hilar lymph node dissection.	26 months (Deceased)	Repeat liver resection for pure large cell neuroendocrine carcinoma of the gallbladder: a favorable outcome	No
Ren Xu(2022) (32)	China	70/F	MiNEN	6	Yes/No	Open cholecystectomy	30 months (Deceased)	Mixed neuroendocrine-non-neuroendocrine neoplasm of the gallbladder: case report and literature review	Yes(papillary adenocarcinoma)
Ren Xu(2022) (32)	China	64/F	MiNEN	2.5×2.5	Yes/No	Cholecystectomy and hepatoduodenal ligament lymph node dissection.	12 months (Alive)	Mixed neuroendocrine-non-neuroendocrine neoplasm of the gallbladder: case report and literature review	Yes(Gallbladder adenocarcinoma)
Yihui Ma(2021) (27)	China	72/M	Large cell neuroendocrine carcinoma of gallbladder with adenocarcinoma and sarcomatoid components	3.5×2.8 ×2.0	No/No	Radical cholecystectomy	2 months (Alive)	Large cell neuroendocrine carcinoma of gallbladder with adenocarcinoma and sarcomatoid components: report of a case	Yes(Gallbladder adenocarcinoma+Sarcoma)
M X Xu(2019) (28)	China	36/F	Large Cell Neuroendocrine Carcinoma of the Gallbladder with well-differentiated adenocarcinoma	4.3×3.5	Yes/No	Laparoscopic cholecystectomy+EP regimen (cisplatin + etoposide)	5 months (Deceased)	Large cell neuroendocrine carcinoma with foci of well-differentiated adenocarcinoma of the gallbladder: report of a case	Yes(Gallbladder adenocarcinoma)
Anisse Tidjane(2021) (5)	Algeria, Africa	68/F	GB-LCNEC	3×2.5× 1.4	Yes/No	Lymphadenectomy of the hepatoduodenal ligament, extended to the retropancreatic and celiac lymph nodes, combined with en bloc resection of the gallbladder and hepatic segments IVb and V+EP regimen (cisplatin + etoposide)	26 months (Alive)	Pure large cell neuroendocrine carcinoma of the gallbladder, is surgical relentlessness beneficial? A case report and literature review	Yes(Breast cancer)

Regarding treatment strategies, our approach was consistent with mainstream literature, emphasizing aggressive surgical intervention as the cornerstone for improving prognosis (31). Most patients with relatively longer survival underwent radical resection (32). In this case, the patient was converted from laparoscopic to open surgery and underwent radical resection for gallbladder carcinoma, which included gallbladder removal, hepatic wedge resection, and hilar lymph node dissection (33).For gallbladder cancer staged pT2b, some guidelines recommend more extensive hepatic resection (segments IVB + V) to improve R0 resection rates (34). However, preoperative imaging could not clearly determine the depth of tumor invasion, and the patient’s active multiple myeloma with impaired bone marrow reserve made extensive hepatic resection a high-risk procedure due to potential postoperative liver dysfunction and infection (35). After comprehensive multidisciplinary team (MDT) evaluation, a conservative hepatic wedge resection was chosen, and pathology confirmed negative margins, demonstrating that this individualized approach achieved R0 resection while ensuring safety. For adjuvant therapy, the standard EP regimen (cisplatin + etoposide) was initially considered but deemed inappropriate due to the patient’s compromised bone marrow reserve and risk of long-term suppression and severe infection (36). After extensive MDT discussion, the patient was started on the CAPTEM regimen (temozolomide + capecitabine), which has shown efficacy in other neuroendocrine tumors with relatively mild toxicity and side effects, making it more suitable for patients with hematologic comorbidities. The absence of recurrence or metastasis during the 6-month follow-up further supports the short-term effectiveness of this individualized treatment strategy (37). However, the generally poor response of high-grade NECs to chemotherapy remains a major challenge limiting long-term survival.

In summary, this case illustrates the extreme rarity, aggressiveness, and diagnostic dependence on pathology of GB-LCNEC, especially when complicated by multiple myeloma (24). Clinical experience confirms that radical surgical resection remains indispensable, while systemic therapy requires precise and individualized strategies tailored to comorbidities and patient-specific conditions to achieve both symptom control and survival extension (38). Future research on GB-LCNEC, particularly in the context of coexisting diseases, should integrate multi-omics approaches to elucidate molecular mechanisms, identify potential therapeutic targets, and promote novel treatment strategies to optimize efficacy and safety (23).

## Conclusion

Gallbladder large cell neuroendocrine carcinoma (GB-LCNEC), as an extremely rare pathological subtype, presents a severe challenge to clinical practice due to its insidious onset, difficulty in preoperative diagnosis, and aggressive biological behavior, leading to generally poor prognosis.

Our findings, combined with a review of the literature, confirm that definitive diagnosis relies almost entirely on postoperative pathology. This is particularly evident through the evaluation of distinctive features such as large cell morphology, high mitotic rate, positive immunohistochemical staining for neuroendocrine markers including INSM1, and a markedly elevated Ki-67 index. In terms of treatment, radical surgery remains pivotal for achieving cure, while effective systemic therapy after surgery is equally critical for delaying recurrence and improving prognosis.

The core value of this case report lies in providing rare and representative data to the globally limited database of gallbladder large cell neuroendocrine carcinoma (GB-LCNEC). It demonstrates how, within the complex clinical context of concurrent multiple myeloma, multidisciplinary team collaboration enabled identification and avoidance of high-risk scenarios associated with standardized chemotherapy regimens, leading to the development of a personalized adjuvant treatment plan tailored to the patient’s actual condition.

Therefore, clinicians should remain vigilant for the possibility of GB-LCNEC when evaluating gallbladder space-occupying lesions and include it in the differential diagnosis. For confirmed cases, highly individualized, multidisciplinary management is essential, especially when complicated by severe comorbidities. Treatment planning must strike an optimal balance between efficacy and safety. Looking forward, further exploration of the molecular mechanisms of this disease and the identification of novel therapeutic targets will be pivotal in improving survival outcomes for these patients.

## Data Availability

The original contributions presented in the study are included in the article/supplementary material. Further inquiries can be directed to the corresponding authors.
